# Effects of L-Carnitine Intake on Exercise-Induced Muscle Damage and Oxidative Stress: A Narrative Scoping Review

**DOI:** 10.3390/nu15112587

**Published:** 2023-05-31

**Authors:** Alberto Caballero-García, David C. Noriega-González, Enrique Roche, Franchek Drobnic, Alfredo Córdova

**Affiliations:** 1 Department of Anatomy and Radiology, Faculty of Health Sciences, GIR Physical Exercise and Aging, University of Valladolid, Campus Los Pajaritos, 42004 Soria, Spain; alberto.caballero@uva.es; 2 Department of Surgery, Ophthalmology, Otorhinolaryngology and Physiotherapy, Faculty of Medicine, Hospital Clínico Universitario de Valladolid, 47003 Valladolid, Spain; dcnoriega1970@gmail.com; 3 Department of Applied Biology-Nutrition, Institute of Bioengineering, University Miguel Hernández, 03202 Elche, Spain; eroche@umh.es; 4 Alicante Institute for Health and Biomedical Research (ISABIAL), 03010 Alicante, Spain; 5 CIBER Fisiopatología de la Obesidad y Nutrición (CIBEROBN), Instituto de Salud Carlos III (ISCIII), 28029 Madrid, Spain; 6 Medical Services Wolverhampton Wanderers FC, Wolverhampton WV3 9BF, UK; docdrobnic@gmail.com; 7 Biochemistry, Molecular Biology and Physiology, Faculty of Health Sciences, GIR Physical Exercise and Aging, University of Valladolid, Campus Duques de Soria, 42004 Soria, Spain

**Keywords:** L-carnitine, muscle damage, oxidative stress

## Abstract

Exercise-induced muscle damage results in decreased physical performance that is accompanied by an inflammatory response in muscle tissue. The inflammation process occurs with the infiltration of phagocytes (neutrophils and macrophages) that play a key role in the repair and regeneration of muscle tissue. In this context, high intensity or long-lasting exercise results in the breakdown of cell structures. The removal of cellular debris is performed by infiltrated phagocytes, but with the release of free radicals as collateral products. L-carnitine is a key metabolite in cellular energy metabolism, but at the same time, it exerts antioxidant actions in the neuromuscular system. L-carnitine eliminates reactive oxygen and nitrogen species that, in excess, alter DNA, lipids and proteins, disturbing cell function. Supplementation using L-carnitine results in an increase in serum L-carnitine levels that correlates positively with the decrease in cell alterations induced by oxidative stress situations, such as hypoxia. The present narrative scoping review focuses on the critical evaluation of the efficacy of L-carnitine supplementation on exercise-induced muscle damage, particularly in postexercise inflammatory and oxidative damage. Although both concepts appear associated, only in two studies were evaluated together. In addition, other studies explored the effect of L-carnitine in perception of fatigue and delayed onset of muscle soreness. In view of the studies analyzed and considering the role of L-carnitine in muscle bioenergetics and its antioxidant potential, this supplement could help in postexercise recovery. However, further studies are needed to conclusively clarify the mechanisms underlying these protective effects.

## 1. Introduction

Supplementation using nutritional products is largely widespread in sports practice. Supplements and sport diets help to prevent nutritional deficiencies, to improve performance and lead to better postexercise recovery [1].

The muscular damage produced by exercise includes structural and functional aspects, which are reflected in a loss of strength, fatigue, myalgia and cramps. Currently, the idea that nutritional supplements, such as L-carnitine, could have beneficial effects in the treatment of muscle damage has been established using different experimental approaches [2]. On the other hand, it is known that exercise produces oxidative stress. This process is associated with the increase in the production of free radicals from mitochondrial activity and the postexercise increase in muscle inflammation [3].

### 1.1. Muscle Damage and Exercise

It is accepted that exercise (endurance or resistance, isometric, eccentric or concentric) is an effective mechanism to increase strength, muscle mass and function. The objective, ultimately, is to improve the quality of life of practitioners (for health purposes) or to improve performance (in sport competition). However, exercise-induced muscle damage (EIMD) and soreness can limit performance [4,5].

During exercise, oxygen demand increases, especially in skeletal muscle, causing a change in blood flow from various organs and systems. These physiological changes produce an increase in oxygen consumption. This results in production of free radicals during and immediately after exercise, leading to an oxidative stress situation. EIMD decreases physical performance [6] and is accompanied by an inflammatory response with infiltration of phagocytes (neutrophils and macrophages). These cells play a key role in the repair and regeneration of tissues. The starting point is the breakdown of damaged proteins and the removal of cellular debris. The release of reactive oxygen species (ROS) occurs during the repair process [7]. Likewise, EIMD is associated with an increase in muscle proteins in blood such as creatine kinase (CK), lactate dehydrogenase (LDH) and myoglobin (Mb). Inflammatory markers such as C-reactive protein (CRP) and inflammatory interleukins (IL), such as IL-1β, IL-6 and tumor necrosis factor-α (TNF-α), increase as well [8,9,10,11]. In addition, EIMD promotes the migration of transcription factors such as nuclear factor-kB (NF-kB) as a response to inflammatory messengers and ROS production [12].

ROS include superoxide, hydroxyl, alkoxyl and peroxyl radicals, but also nonradicals, such as hydrogen peroxide (H_2_O_2_). ROS are closely related to other families of free radicals, such as reactive nitrogen species or RNS (nitric oxide or NO, nitrogen dioxide, peroxynitrite). For this reason, reactive oxygen and nitrogen species (RONS) is a most appropriated terminology. Oxidative stress was initially defined as “an alteration of the prooxidant-antioxidant balance in favor of prooxidants” [13]. This definition is completed with the results of such oxidant unbalance: “oxidative stress leads to an alteration of redox signaling and control and/or to molecular damage” [14]. Inadequate regulation of oxidative stress is correlated with certain physiological and pathological conditions [15,16].

Endogenous sources of RONS are from oxidase activities, such as nicotinamide adenine dinucleotide phosphate (NADPH) oxidase, myeloperoxidase (MPO), lipoxygenase, xanthine oxidase (XO), among others. In addition, RONS are produced as collateral products of mitochondrial electron transport chain [17]. Excessive production of RONS can cause damage to biological molecules such as proteins, carbohydrates, lipids, RNA, and DNA, leading to oxidative tissue damage [18].

There are enzymatic antioxidants such as superoxide dismutase (SOD), catalase (CAT) and glutathione peroxidase (GSH-Px). Nonenzymatic antioxidants include vitamin E, vitamin C, glutathione (GSH) and carotenoids, among others. Antioxidants act to reduce the oxidation potential of RONS, removing or activating redox reactions to inactivate them [19]. In addition, inflammatory processes are linked to oxidative stress. Therefore, the modulation and prevention of these situations during muscle damage and stress could be regulated by taking oral anti-inflammatory or antioxidant supplements [20].

Many nutritional strategies in sports focus on maximizing postexercise recovery and preparing individuals for the next exercise session. Therefore, the potential of certain nutrients and functional foods to decrease EIMD lies in RONS modulation. Actually, this is a very active topic of research.

### 1.2. L-Carnitine

In the human body, L-carnitine (3-hydroxy-4-*N*-trimethylammonium-butyrate) is produced from the amino acids lysine and methionine. Therefore, this compound comes from endogenous synthesis as well as from dietary sources, including ergogenic supplements. Regarding endogenous synthesis, a modified form of the amino acid lysine 6-*N*-trimethyl-lysine (TML) is the starting substrate for carnitine biosynthesis. TML is the product of lysosomal or proteasomal degradation of *N*-trimethylated lysine-containing proteins. In mammals, certain proteins such as calmodulin, myosin, actin, cytochrome c and histones contain *N*-trimethylated lysine residues. These proteins provide TML after proteolytic degradation for carnitine biosynthesis. L-Carnitine is synthesized in the brain, liver and kidney, with muscle being the main tissue reservoir [21,22,23].

L-Carnitine is an essential molecule in cellular energy metabolism due to the acylation of its β-hydroxyl group. The complex carnitine-acyl-CoA is recognized by mitochondrial inner membrane-associated transporters, delivering long-chain fatty acids into the mitochondrial matrix, where they undergo β-oxidation [24]. In mammals, carnitine is considered a “conditionally essential” nutrient, because it can be synthesized by the body, but the main source for the organism comes from diet. It has been estimated that 75% of total body carnitine levels come from diet and only 25% from endogenous synthesis [25]. The main sources of dietary L-carnitine are animal-derived foods, such as red meat, fish and dairy products, as well as nutritional supplements containing highly pure L-carnitine [26]. In addition, the amount of L-carnitine in tissues is conditioned by factors other than diet availability and endogenous synthesis. One of them is renal excretion. In this line, choline supplementation seems to decrease urinary L-carnitine excretion [27]. Moreover, sex differences have been documented, indicating that women have lower circulating levels of carnitine than men [28]. L-Carnitine deficiency is considered when plasma levels are below 20 μmol/L in all age groups. In plasma, 90% of L-carnitine is presented in free form [29]. In normal healthy individuals, skeletal muscle carnitine stores account for 97% of all carnitine in the body with a slow estimated turnover of 105 h [30].

L-carnitine is essential for intermediary metabolism in eukaryotic cells. As mentioned before, the main function is to act as a carrier for the transport of activated long-chain fatty acids from the cytosol to the mitochondrial matrix where β-oxidation takes place. The process is carried out under the control of at least three different proteins: carnitine-palmitoyl-transferase I, acylcarnitine translocase and carnitine-palmitoyl-transferase II. An additional function of L-carnitine is the elimination of RONS [31,32]. As a result, from these functions, L-carnitine modulates acyl-CoA/CoA ratio through the storage of energy in the form of acetylcarnitine and peroxisomal elimination of poorly metabolizable/oxidized acyl groups [33]. The antioxidant action of L-carnitine occurs mainly in the neuromuscular tissue. For this reason, dietary L-carnitine has been used as an essential quaternary ammonium nutrient, exerting favorable effects on energy metabolism and on processes of skeletal muscle remodeling [2,34,35].

In addition, supplementation with L-carnitine produces an increase in serum L-carnitine levels [36]. In this context, there is a significant positive correlation between the increase in serum L-carnitine concentrations and the decrease in the biochemical alteration induced by hypoxia [34,37]. This is explained because increased serum L-carnitine concentrations increase L-carnitine transport across skeletal muscle membrane and the neuromuscular junction. This increase seems to alleviate hypoxia and stimulate acetylcholine synthesis. Consequently, data from recent studies have indicated that sports practitioners may benefit from L-carnitine intake due to increased blood flow and oxygen delivery to muscle tissue, thus reducing hypoxia-related disturbances [34,38]. In this sense, Karlic and Lohninger [26] observed that treatment with L-carnitine modulates the adverse effects of high intensity training by reducing hypoxic damage and accelerating recovery after the stress caused by exercise.

## 2. Objective

The present review focuses on the critical evaluation of the efficacy of L-carnitine supplementation on EIMD, inflammatory and oxidative stress in physically active populations.

## 3. Materials and Methods

This review is focused on the analysis of carnitine supplementation to help in the treatment of postexercise muscular damage. The PICOS question model was used to develop the search and define the inclusion criteria [39].

### Study Analysis and Search Strategy

To conduct the present review, a structured search of SCOPUS, Medline (PubMed) and Web of Science (WOS) databases was performed. The search used related to “Carnitine” AND “muscle damage” OR” muscular damage”, and “Carnitine” AND “exercise stress” OR “Oxidative stress” OR “inflammation”. All search titles and abstracts were separated to identify duplicates and possible missing studies. The inclusion criteria for this review were studies with the aim of identifying a beneficial effect of L-carnitine as a supplement in recovery muscular damage after exercise. We have analyzed studies that were randomized, double-blind controlled, parallel design studies in animal samples or human beings, all in English or Spanish. The “Full search strategy” is presented below.

Titles and abstracts were separated from the search to identify duplicates and missing articles. The suitability of the articles was assessed according to the GRADE concept [40] and the level of evidence [41]. All articles were selected if they had “Moderate” or “High” scientific quality and a gradable grade of evidence from 2 to 2++. Inclusion criteria included studies that aimed to identify a beneficial effect of any form of L-carnitine used alone or as an adjuvant with other products in the recovery from EIMD.

The search was performed according to the Cochrane guidelines for systematic reviews [42]. The Preferred Reporting Items for Systematic Reviews and Meta-Analyses (PRISMA) guidelines were followed [43]. The evaluation was conducted as a scoping review to examine the extent (size), range (variety), and nature (characteristics) of the evidence of the possible effect of L-carnitine on recovery from EIMD. The scoping review also serves to summarize findings from a body of knowledge that is heterogeneous in methods or discipline or identify gaps in the literature to aid the planning and commissioning for future research [44,45]. To analyze the risk of bias, we also used Cochrane guidelines [46]. In view of the domains provided by the tool, we scored those studies satisfying four or more low-risk bias domains as low risk and the remainder as high risk. Two investigators (FD and DC N-G) evaluated the risk of biases independently, with no discrepancies found by a third researcher (AC).

## 4. Results

**Figure 1:**
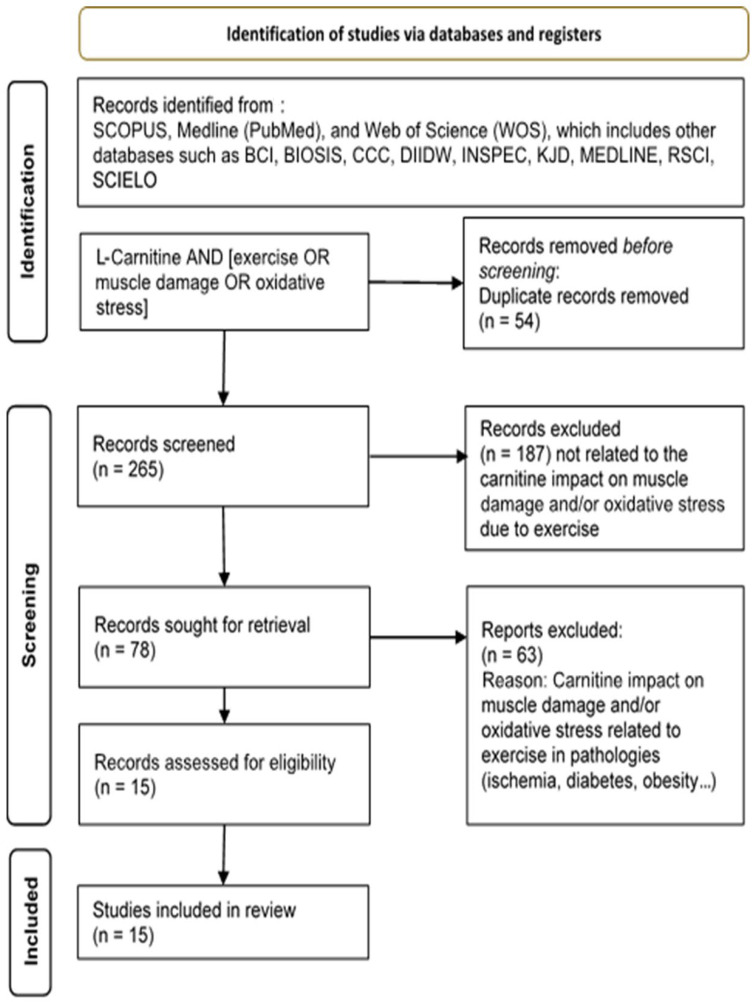
Full search strategy used to develop the systematic review.

**Figure 2:**
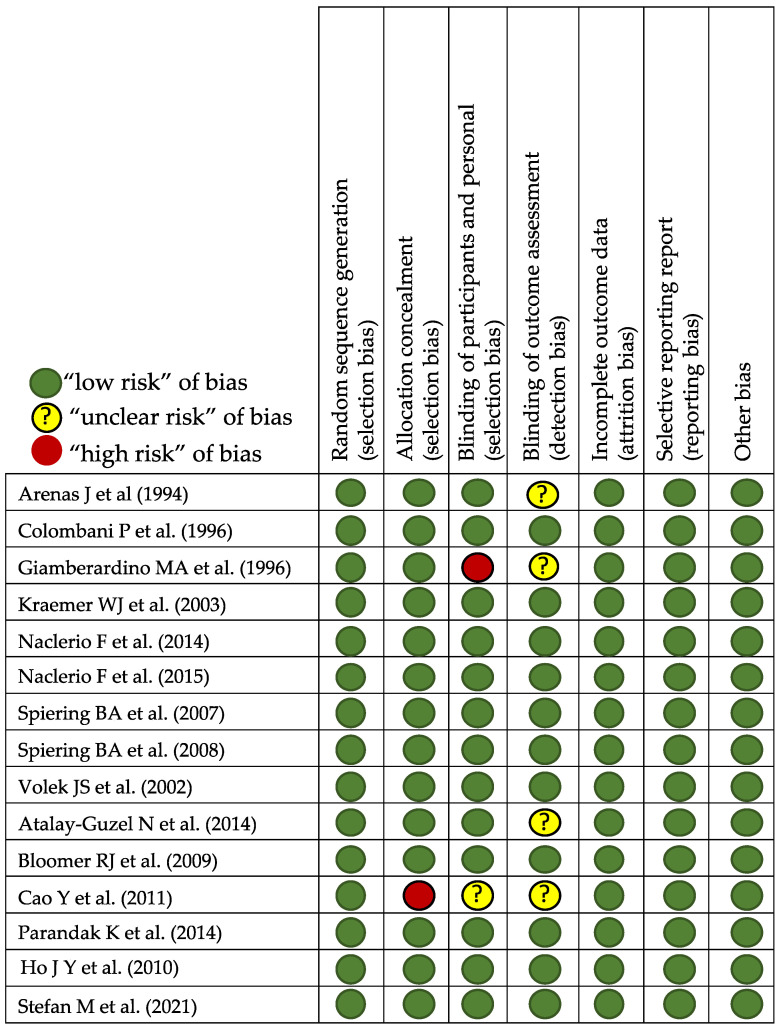
Risk of bias assessment of selected studies [37,47,48,49,50,51,52,53,54,55,56,57,58,59,60].

From the bibliographic search, 78 articles were related to the select descriptors, but only 15 articles met all inclusion criteria (Figure 1). The data of the selected articles are summarized in Table 1. All the evaluated studies contrasted the use of the studied supplement (L-carnitine) vs. placebo, always through oral administration. Five studies evaluated the acute effect on the same day of administration. For the rest, 10 studied the effect of supplement after at least 2 weeks of treatment, the average time being 26 days. In addition, from the 15 studies selected, the impact of L-carnitine supplementation on muscle injury prevention or alteration of myofibrillar structure was observed in 9 studies [37,47,48,49,50,51,52,53,54]. On the other hand, the mitigation of oxidative stress was carried out in five studies [55,56,57,58,59]. Since the response to oxidative stress and muscle injury is a highly associated process, there are only two studies where the improvement from oxidative damage and the decrease in muscle disruption are evaluated in a complementary way. Both studies evaluated L-carnitine administration in healthy individuals of both sexes after muscle-building and power work [59,60]. Altogether, studies on subjects performing a sports activity on a regular basis, competitive or not, are focused on exploring the effect of L-carnitine on the perception of fatigue or delayed onset muscle soreness (DOMS) [49,51,52], muscle injury [37,47,48,49,50,51,52,54] and even intracellular oxygenation levels [37]. Risk of bias of selected articles is shown in Figure 2.

Limitations of the studies are indicated in Table 2.

## 5. Discussion

Acetyl-CoA generation exceeds the capacity of the Krebs cycle when exercise intensity overpasses anaerobic threshold, leading to increased acetyl-CoA, lactate and acetylcarnitine content in skeletal muscle [61,62,63]. These metabolic changes can limit the work capacity of skeletal muscle, since the accumulated acetyl-CoA inhibits the activity of pyruvate dehydrogenase [64]. Under these conditions, the acetyl-CoA/CoA ratio shows a linear correlation with the acetylcarnitine/carnitine ratio [65,66]. Therefore, if the skeletal muscle-free carnitine pool can be increased, the CoA pool should also be increased, which could lead to increased work capacity. Consistent with this concept, Brass et al. [67] described increased force generation and decreased fatigability of skeletal muscle isolated from rat soleus incubated in a buffer containing 10 mmol/L L-carnitine.

Nevertheless, the results of studies trying to assess long-term effects from administration of L-carnitine are somewhat contradictory [68]. In patients undergoing hemodialysis, the administration of L-carnitine for several months shows improved physical performance and trophic effect on skeletal muscle [69]. Similarly, in endurance athletes treated with 2–4 g of L-Carnitine for 4 weeks, similar results were obtained. This observation was associated with an increase in the activity of mitochondrial enzymes, compatible with mitochondrial proliferation [47,70]. However, in a study carried out by Arenas et al. [71] in endurance athletes and sprinters, supplemented with 2 g of oral L-carnitine/day for 4 months, no changes in L-carnitine contents in skeletal muscle at rest were noticed. Nevertheless, a decrease in L-carnitine levels associated with intense muscular exercise was not observed. Therefore, it is important to consider the effects of carnitine supplementation on physical performance, likely acting as a regulator of fuel supply in skeletal muscle, facilitating long-chain fatty acid transport into mitochondria [24,72].

### 5.1. Oxidative Stress in Different Types of Exercise

Actually, it is well established that exercise increases oxidative stress. In this context, in 1988, Gohil et al. [73] observed in trained subjects that cellular GSH levels (nonenzymatic antioxidant) decreased, meanwhile oxidized GSH (GSSG) levels increased concomitantly. From this observation, the presence of oxidative stress was associated with many sport disciplines. In this context, intense aerobic exercise stimulates ROS production [74]. The oxidative stress resulting from aerobic exercise was manifested through increased levels of oxidative damage in lipids, proteins and DNA [75,76,77]. Although it is estimated that aerobic exercise increases oxygen consumption and oxidative stress production during muscle contraction, mitochondria represent only a small fraction of this. The main cause seems to be contractile activity that changes the redox state in muscles to a more oxidative state, reducing the NADH/NAD ratio in mitochondria [78]. However, the oxidative stress that initially occurs postexercise is instrumental to reducing ROS production through the activation of endogenous antioxidant enzymes, such as SOD, GSH-Px and CAT [79,80]. This occurs with moderate exercise intensities, because very demanding exercises with increased ROS formation can impair cellular antioxidant response, provoking infiltration of macrophages and other phagocytes, leading to tissue damage and impaired muscle function [81,82]. Therefore, ROS formation in active skeletal muscle through modulated contractions play an essential role in adaptation to exercise. This adaptation includes an increase in myocellular antioxidant capacity, which helps to reduce ROS levels [79,80,83,84].

Exercise-induced oxidative stress has also been observed following anaerobic exercise. In this sense, several authors [56,85,86,87,88] observed an increase in blood levels of free radicals and oxidative stress markers in subjects performing series of 150 m sprints. In a study carried out by Ammar et al. [89] in which they performed aerobic, anaerobic and combined training, the authors observed that both types of exercise can cause oxidative stress through determination of malondialdehyde (MDA), a marker of lipid peroxidation. They stated that, in both aerobic and anaerobic exercises, a faster response occurs after training, with higher levels of MDA after aerobic training, and with higher levels of SOD and GPX after anaerobic training. These authors concluded that the response to oxidative stress depends on the type of activity, considering intensity and length as main variables [90] of the activity. This observation was supported by Parker et al. [91], who stated that the increase in the intensity of exercise generates more endogenous antioxidant defenses. In this context, the evaluation of oxidative stress using blood tests was confirmed in studies that used muscle biopsies [92,93]. In this context, excessive oxidative stress can lead to impaired physical performance and inadequate recovery of skeletal muscle [94].

### 5.2. Oxidative Stress and L-Carnitine Supplementation

Recently, more studies are being carried out regarding the administration of nutritional supplements in reducing muscle damage and enhancing recovery. In this research area, L-carnitine could act as a regulator of fuel selection in active skeletal muscle leading to an improved contractile function. This might limit potential injury associated with exercise. For this reason, we think that L-carnitine could be considered, not only as an ergogenic aid, but also as a pharmacological treatment in the recovery of athletes suffering from significant muscle damage, depending on the type and length of exercise. However, scarce number of publications directly address this issue. In this review, 15 studies seem to directly explore the response of L-carnitine supplementation to an intense exercise. Only one report was carried out in endurance [58] while the rest were basically resistance exercises. All studies presented a favorable response to the oral administration of 2 g L-carnitine.

As mentioned before, skeletal muscle has several sources of ROS, but mitochondria and cell oxidases appear to be the most relevant sources during exercise [95]. As mentioned before, moderated ROS production can act as mediator of signal transduction pathways, leading to activation of antioxidant muscle response [96]. However, high ROS doses led to imbalance between oxidants and antioxidants favoring oxidants, leading to impaired signaling and redox control and resulting in molecular damage [97]. In vitro studies have demonstrated that increased levels of muscle L-carnitine can modulate oxidative stress by regulating protein synthesis [2]. In this line, Kita et al. [98] showed that L-carnitine supplementation increased plasma concentrations of IGF-1 (insulin-like growth factor-1), activating the corresponding signaling pathway. This increase in IGF-1 seems to be mediated by intramuscular microRNA levels in an animal model [99]. Various studies have reported that IGF-1 not only influences muscle hypertrophy, but also inhibits muscle protein breakdown, responsible for skeletal muscle atrophy [100,101].

In addition, Montesano et al. found that L-carnitine increases key proteins involved in the antioxidant process. This is in line with other studies about the antioxidant activity of L-carnitine [102]. These authors have reported that there is an inverse relationship between efficient β-oxidation of muscle fatty acids and the production of ROS. Through this antioxidant action, L-carnitine could be a good supplement for the prevention and treatment of muscle damage.

### 5.3. Oxidative Stress, Muscle Damage and L-Carnitine Supplementation

As previously discussed, muscle damage is largely the result of the production of ROS, which can cause inflammation and alter cellular functions [103]. High intensity or long duration exercises cause muscle damage, regardless of the eccentric or concentric component of execution. Nevertheless, it is known that eccentric muscle contractions are more impactful on the muscle structure [4,104]. Both in vivo [105] and in vitro [106] studies have shown that mitochondria produce ROS during exercise. A minimal amount of ROS is required for muscle adaptation [107,108]. However, oxidative stress, which results from an increase in muscle ROS concentration, is associated with muscle fatigue during contraction and with postexercise muscle damage [109,110,111,112].

Inflammation produced by exercise due to overstretching of muscle fibers causes damage to the sarcoplasmic reticulum membrane, transverse tubules, or sarcolemma [113]. Both excessive oxidative stress and inflammation can cause damage to DNA, proteins and lipids [114], and an accumulation of advanced glycation end products [115]. The combination of excessive free radical production and the inability of the endogenous antioxidant system to remove them results in delayed recovery and impaired exercise performance. In addition, proinflammatory cytokines such as IL-6 play a key role in the skeletal muscle response, connecting inflammation and oxidative stress [116,117,118,119,120].

When the ROS concentration is too high or sustained over time, a decrease in muscle strength is observed accompanied by muscle fatigue [121]. Therefore, the inflammatory response derived from muscle damage after intense exercise is largely the result of an increase in ROS production [122]. This is confirmed by an increase in the production of proinflammatory cytokines IL-1β, IL-6 and TNF-α, through migration of the transcription factor NF-κβ, the activating protein (AP-1) and cyclooxygenase 2 (COX2) activation [123]. From the point of view of sports performance, the most important effect of muscle damage is decreased muscle function, reducing the ability to generate force [104] and thereby leading to fatigue. However, and despite this, the level of training plays an instrumental role in preventing skeletal muscle damage caused by free radicals [112,124].

An excess of ROS production leads to a reduction in muscle resistance capacity that contributes to fatigue [125]. In this sense, published data are not conclusive and sometimes contradictory. Ohno et al. [126] indicated that SOD levels and activity increased after acute and chronic exercise. Other authors [127,128] presented similar data for changes in CAT activity. Skeletal muscle also produces heat shock or stress proteins (HSP) in response to some forms of contractile activity [129]. These proteins act to prevent tissue damage induced by oxidative stress. In this line, L-carnitine supplementation has been shown to be effective in preventing and attenuating signs of exercise-induced tissue damage [130], likely due to the antioxidant activity of the supplement [131].

### 5.4. L-Carnitine Supplementation and Recovery from Exercise

L-carnitine supplementation seems to improve lipid oxidation, spare muscle glycogen, decrease inflammation and improve exercise performance. The last property seems to occur because L-carnitine supplementation may accelerate recovery from exercise-induced muscle injury [24,132]. In this sense, Dutta et al. [125] observed that the L-carnitine supplement is effective in attenuating the signs of tissue damage induced by exercise. Animal and clinical studies have shown that treatment using L-carnitine positively influences many different mechanisms involved in the pathological loss of skeletal muscle [133].

It is relevant to note that muscle is unable to synthesize L-carnitine due to the lack of γ-butyrobetaine hydroxylase. L-Carnitine is synthesized in the liver, kidney and brain. For this reason, L-carnitine must be transported from plasma to muscle cells. Although there are studies that have shown an increase in plasma L-carnitine after supplementation, only few studies have shown a subsequent increase in muscle [134,135]. Muscle uptake seems to be a long process, as observed by Wall et al. [136], who obtained increases in muscle L-carnitine after long-term supplementation in subjects with hyperinsulinemia.

The beneficial effects of L-carnitine in exercise recovery have been observed in healthy subjects and in others suffering certain pathologies [137]. However, results have been contradictory. In this line, Swart et al. [138] measured exercise performance in marathon runners after 6 weeks of L-carnitine supplementation and found a positive impact on maximum treadmill running speed, as well as maximum oxygen consumption (VO_2_max). Vecchiet et al. [139] also observed that L-carnitine supplementation significantly increased VO_2_max and starting power. In contrast, Krähenbühl [140] analyzed the impact of 3 months of L-carnitine supplementation on physical performance and found no improvement.

On the other hand, Dubelaar et al. [141] observed that the administration of L-carnitine increased muscle contractile force by 30% accompanied by an increase in blood flow in a dog model. These changes were associated to an increase in L-carnitine levels in plasma, but with no significant increase in muscle. These authors hypothesized that L-carnitine exerts the effect on the vascular cells surrounding muscle and thereby increasing oxygen delivery.

In this line, Giamberadino et al. [49] proposed an alternative mechanism to explain the effect of L-carnitine in exercise recovery. They observed, following L-carnitine supplementation (3 g/day for 3 weeks) in healthy untrained men, a reduction in circulating CK and DOMS compared to placebo. The authors stated that the effect was due to the vasodilatory effect of L-carnitine which, according to their hypothesis, reduces hypoxic stress, an action similar to some vasodilators. Recently, we observed that supplementation with NO precursors favors vasodilation, allowing for high bioavailability of nutrients and hormones to the muscles, thus helping physical performance [142].

## 6. Conclusions

The presented studies analyzed the role of L-carnitine supplementation in muscle bioenergetics and its antioxidant potential in physically active individuals. In this context, L-carnitine supplementation could be an ergogenic aid, helping in muscle damage and recovery, particularly in conditions of L-carnitine deficiency. However, further studies are needed to conclusively clarify the mechanisms underlying these protective effects.

## Figures and Tables

**Table 1:** Studies evaluating the effect of L-carnitine on muscle damage and oxidative stress related to exercise.

Reference		Molecule/s	Daily Dosage	Route	Days	Placebo/Control	*n*	Type of Subjects	Age (Years)	Tests	Impact on Resolution
Arenas J et al., (1994)	[47]	L-carnitine	2 g	Orally	28	P and C	8P/8S/ 22 C	High level male athletes	28 ± 7	Histology (muscle biopsies)	⊕ ↑ pyruvate dehydrogenase, ⊕ ↑ in the activities of complexes I, III and IV of the respiratory chain.
Colombani P et al., (1996)	[48]	L-carnitine	4 g (2 + 2)	Orally	1	P	10	High level male athletes	36 ± 3	Blood analysis after marathon race	↔ marathon running time, ↔ plasma concentrations of carbohydrate metabolites; ↔ fat metabolites, ↔ hormones (insulin, glucagon, cortisol), ↔ enzyme activities (CK).
Giamberardino MA et al., (1996)	[49]	L-carnitine	3 g	Orally	21	P	6	Healthy males	26 ± 4	Blood analysis after eccentric effort, VAS	⊕ ↓ pain, ↓ tenderness and ↓ CK release.
Kraemer WJ et al., (2003)	[50]	L-carnitine + L-tartrate	2 g	Orally	21	P	10	Resistance-trained males	26 ± 2	Blood analysis after resistance effort, MRI	⊕ ↓ exercise-induced muscle tissue damage, ↑ IGFBP-3.
Naclerio F et al., (2014)	[51]	L-carnitine + L-tartrate + MI	3 g	Orally	1	P	16	Amateur soccer male players	24 ± 4	Blood analysis after intermittent repeated sprint test, RPE	⊕ perception of fatigue, ↓ myoglobin, ↔ intermittent performance, ↔ inflammatory or immune function.
Naclerio F et al., (2015)	[52]	L-carnitine + L-tartrate + MI	3 g	Orally	1	P	10	Team sport male players	25 ± 4	Blood analysis after intermittent repeated sprint test, RPE	⊕ ↓ myoglobin, ↓ CK, ↔ perception of fatigue, ↔ sprint performance, ↔ inflammatory or immune function.
Spiering BA et al., (2007)	[37]	L-carnitine + L-tartrate	1 or 2 g	Orally	21	P	8	Resistance-trained male	22 ± 3	Blood analysis after resistance effort.	⊕ ↓ hypoxanthine, xanthine oxidase, myoglobin, and perceived muscle soreness.
Spiering BA et al., (2008)	[53]	L-carnitine + L-tartrate	2 g	Orally	23	P	9	Resistance-trained male	25 ± 6	Blood analysis after resistance effort.	⊕ ↓ muscle oxygenation during upper arm occlusion and following each set of resistance exercise.
Volek JS et al., (2002)	[54]	L-carnitine tartrate	2 g	Orally	21	P	10	Resistance-trained male	24 ± 2	Blood analysis after resistance effort, MRI	⊕ ↓ markers of purine catabolism (hypoxanthine, xanthine oxidase, and serum uric acid) and ↓ circulating muscle proteins (myoglobin, fatty acid-binding protein, and creatine kinase). ↓ muscle disruption from MRI scans.
Atalay Guzel N et al., (2014)	[55]	L-carnitine	3 or 4 g or P	Orally	1	P	13	Healthy males	17–19	Maximal exercise test	⊕ ↑ GSH and NO, ↓ TBARs
Bloomer RJ et al., (2009)	[56]	Propionyl L-carnitine	1 or 3 g or P	Orally	56	P	32	Healthy males and females	27 ± 2, P 26 ± 2, 1 g 27 ± 2, 3 g	Aerobic–anaerobic exercise testing	Both aerobic and anaerobic power testing increase oxidative stress to a similar extent. ⊕ ↓ MDA, but little impact on exercise-induced oxidative stress biomarkers.
Cao Y et al., (2011)	[57]	L-carnitine	2 g	Orally	1	U	12	Healthy males and females	28 ± 5	Blood analysis	⊕ ↑ SOD, ↑ GSH-Px, ↑ catalase and ↑ TAC following the first 3,5 h post-administration.
Parandak K et al., (2014)	[58]	L-carnitine	2 g	Orally	14	P	21	Healthy males	22 ± 1	Blood analysis after endurance exercise	⊕ ↑ TAC, ↓ MDA-TBARS, CK, and LDH 24 h after exercise.
Ho JY et al., (2010)	[59]	L-carnitine	2 g	Orally	24	P	18	Healthy males and females	45 ± 5, m 52 ± 5, f	Blood analysis after resistance effort	⊕ ↓ biochemical markers of purine metabolism, ↓ MDA, ↓ muscle tissue disruption (myoglobin, CK), ↓ muscle soreness.
Stefan M et al., (2021)	[60]	L-carnitine tartrate	2 g	Orally	35	P	73	Healthy males and females	39 ± 1, m 41 ± 2, f	Blood salivary analysis, soreness scale	⊕ ↑ SOD, ↓ perceived recovery and soreness, ↓ CK.

⊕: effective; ↑: higher or improved; ↓: lower; ↔: similar than the control group; C: control/inactive group; CK: creatine kinase; GSH: glutathione; GSH-Px: glutathione peroxidase; IGFBP-3: insulin-like growth factor-binding protein-3; LDH: lactate dehydrogenase; MDA: malondialdehyde; MI: multi-ingredient (106 g carbohydrates, 14.5 g whey protein, 5 g glutamine); MRI: magnetic resonance imaging; NO: nitric oxide; P: placebo group; RPE: rate of perceived exertion; S: supplemented group; SOD: superoxide dismutase; TAC: total antioxidant capacity; TBARs: thiobarbituric acid-reactive substances; U: unknown; VAS: visual analogue scale for pain.

**Table 2:** Some limitations of studies referred in Table 1.

Reference	Limitations
Arenas et al. [47] Colombani et al. [48] Giamberardino et al. [49] Parandak et al. [58]	Small sample size conducted in endurance athletes, limiting the extension to other populations.
Spiering et al. [37] Kraemer et al. [50] Spiering et al. [53] Volek et al. [54]	Small sample size conducted in resistance male athletes, limiting the extension to other populations.
Nacleiro et al. [51] Nacleiro et al. [52] Atalay Guzel et al. [55]	Small sample size conducted in intervallic athletes, limiting the extension to other populations.
Cao et al. [57]	Small sample size conducted in healthy individuals, no representative of a broader population.
Ho et al. [59]	Small sample size conducted in middle-aged individuals, no representative of a broader population.
Cao et al. [57] Parandak et al. [58] Ho et al. [59]	No control group.
Arenas et al. [47] Colombani et al. [48] Kraemer et al. [50] Nacleiro et al. [51] Bloomer et al. [56]	The study does not investigate the role of L-carnitine supplementation on postexercise recovery.
Spiering et al. [37] Arenas et al. [47] Colombani et al. [48] Kraemer et al. [50] Nacleiro et al. [51]	The study does not investigate the role of L-carnitine supplementation on postexercise oxidative stress.
Colombani et al. [48]	The study was conducted in a field setting, limiting the control of other variables.
Giamberardino et al. [49] Stefan et al. [60]	Only data from CK release, but no data from other markers of postexercise muscle damage or oxidative stress were presented.
Atalay Guzel et al. [55] Bloomer et al. [56]	The study did not provide data on muscle damage.
Cao et al. [57]	Short duration and single dose administration. This limits the possibility to draw long-term conclusions.

Abbreviations used: CK—creatine kinase.
